# Adverse childhood experiences, recent negative life events, and non-suicidal self-injury among Chinese college students: the protective role of self-efficacy

**DOI:** 10.1186/s13034-022-00535-1

**Published:** 2022-12-03

**Authors:** Zixun Chen, JinWen Li, JinMeng Liu, Xia Liu

**Affiliations:** 1 School of Education Science, Nanjing Normal University, No. 122 Ninghai Road, Gulou District, Nanjing, 210097 Jiangsu China; 2 Institute of Developmental Psychology, Beijing Normal University, No. 19 Xinjiekouwai Street, Beijing, 100875 China

**Keywords:** Non-suicidal self-injury, Self-efficacy, Adverse childhood experiences, Recent negative life events, College students

## Abstract

**Background:**

Non-suicidal self-injury (NSSI) is a severe health problem closely related to adverse childhood experiences (ACEs). However, the underlying mechanisms by which ACEs may affect NSSI are largely unknown. Self-efficacy (NSSI-SE) and recent negative life events (RNLEs) may play important roles in this relationship. This study aimed to clarify the relationship between ACEs and NSSI among college students by examining the role of self-efficacy (NSSI-SE) and RNLEs in this process.

**Method:**

Relevant self-report questionnaires were used to evaluate ACEs, RNLEs, NSSI-SE, and NSSI. A questionnaire of 1036 Chinese undergraduates (*M*_age_ = 19.65, 28.9% males, 71.1% females) was collected in a cross-sectional manner. The associations between ACEs, RNLEs, NSSI-SE and NSSI were assessed using Pearson correlation analyses. Then, hierarchical multiple linear regressions were used to analyze the effects of ACEs and RNLEs on NSSI, as well as the protective effect of NSSI-SE on the above relations.

**Results:**

NSSI was associated with both ACEs and RNLEs. ACEs and RNLEs could directly increase the risks of participating in NSSI, and the effects of ACEs and RNLEs on NSSI were independent without an interactive effect. NSSI-SE buffered the relationship between ACEs and NSSI, as well as between RNLEs and NSSI. Compared to individuals with a low level of NSSI-SE, ACEs and RNLEs were not significantly associated with NSSI in persons with a high level of NSSI-SE.

**Conclusion:**

NSSI-SE may buffer the effect of ACEs and RNLEs on NSSI, indicating that future interventions can be enhanced by targeting NSSI-SE among college students with ACEs or RNLEs to prevent their engagement in NSSI.

## Introduction

Non-suicidal self-injury (NSSI) is widely recognized as a significant public health and societal concern that can bring challenges and disadvantages to individuals’ development [1]. NSSI refers to an act of direct and deliberate destruction of body tissue without the intent to commit suicide and is distinguished from reckless or careless behavior that may indirectly result in bodily harm [2, 3]. Such behavior frequently occurs among university students [4, 5]. For instance, a recent study found that nearly 22% of Chinese college students reported having engaged in NSSI in the past year [6].

Among the studies on the factors associated with NSSI, one consistent factor that has been identified as a significant predictor of NSSI is the occurring adverse childhood experiences (ACEs) [7–9]. Related studies have found that individuals with a history of ACEs are more likely to engage in NSSI [10–13]. However, these studies mainly focus on inpatients and adolescents [8, 14, 15], and the investigation of this topic among college students needs to be strengthened. In addition, recent negative life events (RNLEs), which refer to the experience of stressful events in the last year, can be theorized to have a similarly prominent role as a main proximal risk factor for NSSI, and they may play a moderating role in the relationship between ACEs and NSSI. Moreover, the self-efficacy to avoid NSSI (NSSI-SE) is an important protective factor in the relationships between ACEs, RNLEs, and NSSI [16–18]. For college students with different levels of NSSI-SE, the effects of ACEs and RNLEs on NSSI may be inconsistent. At present, there is still no research on this issue.

Investigating protective and risk factors simultaneously can help formulate targeted interventions for college students’ NSSI, especially through the exploration of protective factors, which can help high-risk groups avoid NSSI. The current study, therefore, aimed to explore the specific effect of ACEs and RNLEs on NSSI in college students and investigate the potential buffering effect of NSSI-SE of NSSI in this process. The focus of this study is whether NSSI-SE can effectively protect college students who have suffered from ACEs and RNLEs.

### Adverse childhood experiences and NSSI

Adverse childhood experiences are defined as a series of moderate to severe stressful encounters occurring in childhood, including emotional, sexual, or physical abuse; parental incarceration; neglect; divorce; parental psychopathology; and parental separation [19]. Numerous investigations have found that a history of ACEs is a significant determinant of NSSI in adolescents as well as young adults [7, 20], and as many as 80% of those who engage in NSSI have a history of ACEs [21].

According to the model of difficulties in emotion regulation, NSSI is considered a maladaptive means to regulate emotions after suffering from negative events [22]. In the past literature, ACEs were recognized as a series of typical negative events [23, 24]. Based on this opinion, young adults and college students with a history of ACEs, are likely to have trouble coping with these past negative events. Thus, individuals with a history of suffering from negative events (i.e., ACEs) may engage in NSSI as a method to regulate their affect and emotions [25, 26]. The theory hypothesizes that engaging in NSSI derives from a need to control past experiences of trauma and current anger and pain related to such experiences that cannot be expressed verbally or through other means [26]. Both cross-sectional and longitudinal studies support the function of emotional regulation as an explanation for NSSI among young adults and teenagers with a history of ACEs (a series of classic childhood negative events [8, 23, 24, 27]) [28–30]. In addition, systematic reviews and meta-analyses clarify the predictive effect of ACEs on NSSI [10, 11] and show that people with a history of ACEs are more likely to engage in NSSI.

### Recent negative life events and NSSI

Recent negative life events (RNLEs) are defined as psychosocial stressors and include a range of distressing life events that may occur in a young person’s daily life, such as “having conflicts with members of one’s family,” “being misjudged by friends,” and “failing examinations.” [31] According to previous research, recent negative life events are important risk factors for NSSI. For instance, some studies reveal that RNLEs are positively related to children’s and adolescents’ depression and NSSI [32–34]. Life stress brought on by recent negative life events has been identified to play a unique role in the prevalence of NSSI, whereby people with more life stress are vulnerable to participating in NSSI [35]. However, despite some researchers pointing out that the history of RNLEs is an important factor in studying NSSI, existing research mainly examines the relationship in adolescents, while few have clarified the relationships of NSSI in college students and young adults with RNLEs. To address this gap, one of the goals of the present study was to examine the degree to which RNLEs influence NSSI among college students.

Another concern is that the history of RNLEs may play an important role in the relationship between ACEs and NSSI. On the one hand, the concept of stress sensitization suggests that RNLEs perpetuate the negative impact of ACEs and contribute to the onset and recurrence of psychopathology symptoms [36]. Thus, ACEs and RNLEs may interact to affect NSSI. On the other hand, some studies also show that early and later-life stressors may make independent contributions to health later in life, such as in terms of inflammation, daily negative affect, and depression [37–39]. In other words, ACEs and RNLEs may independently affect adult mental health outcomes. Taken together, the relationships between ACEs, RNLEs, and psychological outcomes (i.e., NSSI) appear to be complex, and more empirical research is needed to clarify the effect of ACEs and RNLEs on NSSI among Chinese college students.

### The buffering effect of self-efficacy

Given the elevated prevalence of NSSI among university students, it is important to determine possible protective aspects linked to a reduction in the incidence of NSSI. The transtheoretical model of change (TTM) provides a new direction for the therapeutic treatment of NSSI [40, 41]. The TTM constitutes a theoretical approach to intentional behavior change that comprises four extensive domains, i.e., stages of change, decisional balance, the change process, and self-efficacy [42]. The domain of self-efficacy is essential for effective intervention. Regarding NSSI, self-efficacy is generally redefined as the assessment of an individual’s ability to resist self-injury [43]. Self-efficacy to avoid NSSI (NSSI-SE) provides a new perspective into specific NSSI-related cognitions. The proposed NSSI-SE refers to considering enhancing an individual’s specific confidence in their ability to avoid NSSI behaviors [43, 44]. In other words, NSI-SE is an element proposed by clinicians to try to provide specialized interventions for people with NSSI. This domain is integrated from Bandura’s social cognitive theory (SCT) and includes two parts: confidence and temptation [45]. Confidence represents an individual’s situation-specific confidence in his or her ability to cope with high-risk situations without reverting to undesired behavior. Temptation refers to an individual’s rating of the strength of his or her desire to engage in a certain behavior (NSSI) when amid a difficult situation. Temptations can be internal, taking the form of negative affect or emotional distress, or external, taking the form of triggering relationships or external stimuli [46, 47].

A few empirical studies based on the TTM have established that NSSI-SE is lower in persons who commit NSSI and that diminished confidence in one’s capacity to cope with distressing events might trigger the onset of NSSI and result in maintaining it over time [48, 49]. Furthermore, social self-efficacy, a belief in one’s potential to manage social occurrences, protects against NSSI risks resulting from experiencing a perceived injustice in a family setting [50], implying that NSSI-SE may play a protective role for teenagers and young adults who experience adversity and distressing life events. Therefore, it is very important to consider the potential moderating effect of NSSI-SE when conducting in-depth research on the link between ACEs, RNLEs, and NSSI in undergraduates. For vulnerable college students who have encountered stressful ACEs and RNLEs, investigating NSSI-SE to prevent NSSI may be significant in identifying effective strategies for NSSI prevention.

### Current study

**Fig. 1:**
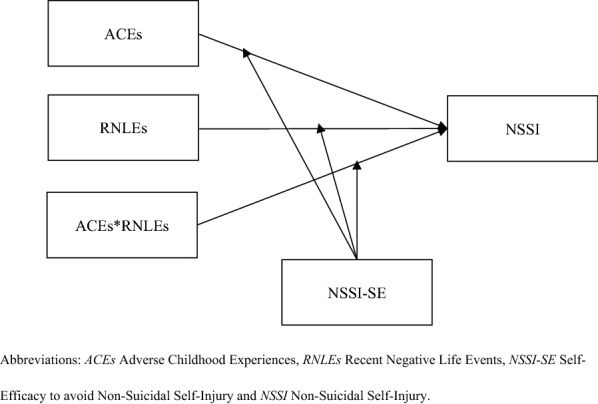
Pathway diagram of the hypotheses

Given the limitations of previous studies, we tested the role of ACEs and RNLEs in affecting NSSI and especially focused on the protective effect of NSSI-SE in a sample of Chinese college students. Therefore, We hypothesized that the relationship between ACEs and NSSI would be stronger for individuals with more RNLEs (ACEs × RNLEs). Furthermore, we hope to investigate the buffering effect of NSSI-SE in the relationship between ACEs and NSSI (ACEs × NSSI-SE), as well as between RNLEs and NSSI (RNLEs × NSSI-SE). Finally, we examined whether the interactive association between ACEs and RNLEs in affecting NSSI differs with levels of NSSI-SE (ACEs × RNLEs × NSSI-SE), i.e., whether NSSI-SE can reduce the risk of NSSI in groups with a history of ACEs and RNLEs. The relationships among all variables are shown in Fig. 1.

## Methods

### Participants and procedure

Subjects were enrolled via an internet research source, *Sojump,* and each participant volunteered to participate in this study without payment. A total of 1109 students from universities across China completed the online questionnaire. A total of 1064 valid responses that met the screening requirements of participants were obtained, resulting in a valid response rate of 95.94%. Since the questionnaire of this study was published on a public and open online questionnaire collection data platform, many college students who are not yet 18 years old filled in the questionnaire after seeing the recruitment link, for ethical consideration, 28 students’ responses were excluded due to the violation of age limit. Of the final 1036 participants, 737 were female (71.1%), and 299 (28.9%) were male, with a mean age of 19.65 years (*SD* = 1.23, range 18–25 years). The participants included 203 (19.6%) first-year students, 580 (56.0%) second-year students, 168 (16.2%) third-year students, 74 (7.1%) fourth-year students and 11 (1.1%) fifth-year college students. The participants majored in a wide range of subjects, including the arts, the humanities, the social sciences, the natural sciences, and medicine.

The questionnaire was preceded by the distribution of information regarding the purpose, scope, and content of the survey. The students were also informed that their participation was voluntary and that the data collected would be kept completely confidential. All of the subjects agreed to participate without further questions. Data were collected from an open survey platform, and each participant took approximately 35 min to complete the questionnaire. The study was granted approval by the institutional review board of the author’s university and complied with the Declaration of Helsinki regarding the use of human subjects.

### Measures

ACEs were assessed using the Chinese version of the Adverse Childhood Experiences International Questionnaire (ACE-IQ) [51, 52], which has been established to show good reliability coupled with validity in China [53]. The 32-item measure explores exposure to four domains of ACEs: neglect, family dysfunction, abuse, and violence. Each domain includes several categories. We used 28 items from all categories, with the exception of exposure to collective violence/war (four items). Participants were asked to respond to the 28 questions on the basis of their encounters during their first 16 years of life. Each item was then constructed as a dummy variable with a score of “0” assigned if the participant had never experienced it and a code of “1” assigned if the participant had experienced it during the first 16 years of life. The overall number of ACE items to which the subject was “exposed” was summed to construct an ACE score ranging between 0 and 28. This study focused on the findings of subjects’ overall ACE exposure (ACE score). Overall, the Cronbach’s alpha coefficient of the ACE-IQ (ACE score) was measured as 0.84.

RNLEs were investigated by the Adolescent Self-Rating Life Events Checklist (ASLEC) [31, 54], which is utilized to explore the impact of negative life events on teenagers (including undergraduates) over the previous 12 months. The ASLEC includes 26 items linked to five dimensions: study stress, punishment, relationship pressure, loss, and healthy adaptation. The checklist uses a six-point scale (1 = no negative life events to 6 = negative life events with serious impact). The higher a score is, the more distressing the associated negative life event was. In this study, the Cronbach’s alpha coefficient of the scale was measured as 0.92.

Self-efficacy to avoid NSSI (NSSI-SE) was explored via an 11-item questionnaire adapted from the scale measuring NSSI-SE to avoid committing non-suicidal self-injury [43]. Participants reported on a 5-point scale ranging from 1 (very unfit) to 5 (very fit) whether they believed they could resist engaging in NSSI in certain situations based on statements such as “I feel like I can accept myself,” “I need to relieve inner pressure or need a release,” and “I have easy access to (self-harming) tools.” The scale included three subscales, for “confidence,” “emotion regulation temptations,” and “reinforcing temptations.” The greater the total score is, the stronger a student’s level of NSSI-SE to resist NSSI is. The Cronbach’s α of the current sample was measured as 0.89.

NSSI was tested using the 18-item Adolescents Self-Harm Scale (ASHS) [55]. This instrument explores the degree to which persons repeatedly or deliberately self-harm via diverse methods (e.g., hitting, skin-cutting, or stabbing) without harboring suicidal intentions. Two dimensions of each of the 18 items were evaluated. The first evaluated the number of times a participant deliberately adopted this method to self-harm at any time previously (frequency) and used a four-point scale (0 = has not happened, to 3 = has happened more than five times); the second item evaluated the extent to which a previous act had hurt the participant’s body at any time (degree) on a five-point scale (0 = no harm to the body, to 4 = extreme severe harm to the body). Each item’s score was equal to the frequency multiplied by the extent, and the overall score (summed score across all items) was employed to compute the overall extent of NSSI. The higher a participant’s total score was, the higher their degree of NSSI was. The Cronbach’s α for ASHS was measured as 0.92.

Finally, subjects were asked to report their age, gender, grade, and study major and their parents’ education level (6 levels from 1 = elementary school graduate or below to 6 = postgraduate degree graduate or above). Subjective socioeconomic status was explored via the MacArthur ladder scale of subjective social status (10 steps) [56].

### Data analysis

All data analyses were implemented in SPSS 24.0. Since all the measurements were based solely on self-reporting, common method variance could have influenced the associations between the variables. Harman’s single-factor test was performed prior to data analyses [57]. The results show that 25 factors generated eigenvalues greater than 1.0 (68.8% of the explained variance). At each evaluation, the first factor was responsible for 16.33 (less than 40%) of the variance, implying that the common method variance was insignificant.

In the current study, NSSI data were highly skewed (skewness = 6.59, kurtosis = 55.85), and there was an inhomogeneity of variance between the male and female groups (*F* = 5.79, *p* < 0.05), so the data did not fit the preconditions of the independent sample *t* test. Then, since gender differences have been found in previous studies on NSSI [58–60], the Mann‒Whitney *U* test was adopted to investigate whether the gender difference exists in our study [61]. The NSSI score was normally transformed to meet the requirements of subsequent analyses. Since some participants reported an NSSI score of 0, the natural logarithm was taken based on the addition of a constant of 1 to the NSSI score to form a new NSSI score with an approximately normal distribution (skewness = 2.15, kurtosis = 4.12) to meet the requirements of subsequent correlation analyses and linear regressions [62–64]. Then, Pearson correlation analyses examined correlations between all study variables. Referring to the analytical methods of existing studies [65–69], we conducted a hierarchical multiple regression to test our hypotheses regarding the specific effects of ACEs, RNLEs, and NSSI-SE in predicting NSSI.

The model explored the potential protective role of NSSI-SE in the relationship between ACEs, RNLEs, and NSSI. In Step 1 of this regression, some sociodemographic variables were entered. In Step 2 of this model, ACEs, RNLEs, and NSSI-SE were entered into the analysis. Interactions (ACEs multiplied by RNLEs, ACEs multiplied by NSSI-SE, and RNLEs multiplied by NSSI-SE) were entered in Step 3. Finally, a three-way interaction variable (ACEs multiplied by RNLEs multiplied by NSSI-SE) was entered in Step 4. For any significant interactions, simple slope tests were performed afterward. All continuous variables were standardized (***z*** scores) prior to analysis to overcome multicollinearity by using NSSI scores after natural log transformation to ensure that the dependent variable was normally distributed.

## Results

### Preliminary analyses

Of the 1,036 participants, 24.4% (*n* = 253) reported having engaged in one or more episodes of NSSI over the past 12 months. The Mann‒Whitney *U* test was utilized to test gender differences in NSSI levels. The data show that the level of NSSI in males (*M* = 2.05, *SD* = 6.81) was not significantly different from that in females (*M* = 1.48, *SD* = 5.02, *p* > 0.05). Since no gender difference was found in the level of NSSI, subsequent analyses were carried out among all participants.

Then, Pearson correlation analyses were employed to test the bivariate correlations of the variables.

As shown in Table 1, gender was negatively associated with ACEs (*r* = − 0.12, *p* < 0.001) and RNLEs (*r* = − 0.09, *p* < 0.01), which means that males reported more ACEs and RNLEs than females. Age was positively associated with ACEs (*r* = 0.15, *p* < 0.001) and RNLEs (*r* = 0.08, *p* < 0.05), while age was negatively associated with NSSI-SE (*r* = -0.09, *p* < 0.01). ACEs (*r* = 0.29, *p* < 0.001) and RNLEs (*r* = 0.28, *p* < 0.001) were significantly positively associated with NSSI, NSSI-SE was significantly negatively associated with NSSI (*r* = − 0.31, *p* < 0.001). ACEs (*r* = − 0.28, *p* < 0.001) and RNLEs (*r* = − 0.39, *p* < 0.001) were inversely associated with NSSI-SE. Subjective socioeconomic status was negatively associated with RNLEs (*r* = − 0.11, *p* < 0.01) and positively associated with NSSI-SE (*r* = 0.10, *p* < 0.01). Fathers’ education levels were positively associated with NSSI (*r* = 0.09, *p* < 0.01). Mother’s education levels were positively associated with NSSI (*r* = 0.06, *p* < 0.05). Because age, subjective socioeconomic status, and parents’ education levels are important demographic variables closely related to the development of individual behavioral outcomes [5, 33, 70], we treated these as covariates in the downstream analysis.

**Table 1:** Means, standard deviations, and correlations among all study variables (*N* = 1036)

Variable	Range	1	2	3	4	5	6	7	8	9
1 Gender	0–1	–								
2 Age	18–25	− 0.09^**^	–							
3 SSES	1–10	0.05	0.03	–						
4 Father’s education level	1–6	− 0.06	0.08^**^	0.25^***^	–					
5 Mother’s education level	1–6	− 0.03	0.06^*^	0.19^***^	0.68^***^	–				
6 ACEs	0–25	− 0.12^***^	0.15^***^	− 0.04	− 0.02	− 0.01	–			
7 RNLEs	1–6	− 0.09^**^	0.08^*^	− 0.11^**^	− 0.04	− 0.05	0.44^***^	–		
8 NSSI-SE	1–5	0.01	− 0.09^**^	0.10^**^	− 0.01	0.03	− 0.28^***^	− 0.39^***^	–	
9 NSSI	0–68	− 0.05	0.04	− 0.04	0.09^**^	0.06^*^	0.29^***^	0.28^***^	− 0.31^***^	–
*M* ± *SD*	–	–	19.65 ± 1.23	4.98 ± 1.55	2.74 ± 1.32	2.42 ± 1.26	4.99 ± 4.44	1.97 ± 0.68	4.08 ± 0.80	1.64 ± 5.60

***Gender:*** 0 = male; 1 = female. ***Range:*** the minimum and maximum scores achieved in the sample

*M* mean, *SD* standard deviation, *SSES* Subjective Socioeconomic Status, *ACEs* Adverse Childhood Experiences, *RNLEs* Recent Negative Life Events, *NSSI-SE* Self-Efficacy to Avoid Non-Suicidal Self-Injury and *NSSI* Non-Suicidal Self-Injury

^*^p < 0.05

^**^p < 0.01

^***^p < 0.001.

### The moderating role of self-efficacy (NSSI-SE) in associations of NSSI with ACEs and RNLEs

Table 2 shows that ACEs (*β* = 0.19, *p* < 0.01) and RNLEs (*β* = 0.12, *p* < 0.01) significantly positively predicted NSSI, and NSSI-SE (*β* = − 0.26, *p* < 0.001) significantly predicted NSSI. Additionally, the two-way interaction between ACEs and NSSI-SE was significant in predicting NSSI (*β* = − 0.12, *p* < 0.01). Furthermore, the two-way interaction between RNLEs and NSSI-SE proved significant in predicting NSSI (*β* = − 0.10, *p* < 0.01). Finally, the two-way interaction of ACEs × RNLEs and the three-way interaction of ACEs × RNLEs × NSSI-SE did not prove significant. The full set of predictors explained 23.1% of the variance in NSSI.

**Table 2:** Results of hierarchal multiple regression analyses predicting NSSI by ACEs, RNLEs, NSSI-SE and their interactions

Variable		*NSSI* (*N* = 1036)			
		ΔR^2^	*β*	*SE*	*t*
Step 1 (control variables)	Age	0.02^***^	0.06	0.02	1.80
	SSES		− 0.10	0.02	− 3.36^**^
	Father’s education level		0.08	0.03	1.76
	Mother’s education level		0.04	0.03	0.99
Step 2	ACEs	0.18^***^	0.19	0.03	6.05^***^
	RNLEs		0.12	0.03	3.80^***^
	NSSI-SE		− 0.26	0.03	− 8.37^***^
Step 3	ACEs × RNLEs	0.03^***^	− 0.03	0.02	− 0.84
	ACEs × NSSI-SE		− 0.12	0.03	− 3.39^**^
	RNLEs × NSSI-SE		− 0.10	0.02	− 2.94^**^
Step 4	ACEs × RNLEs × NSSI-SE	< 0.001	0.01	0.02	0.12

^*^p < 0.05

^**^p < 0.01

^***^p < 0.001

**Fig. 2:**
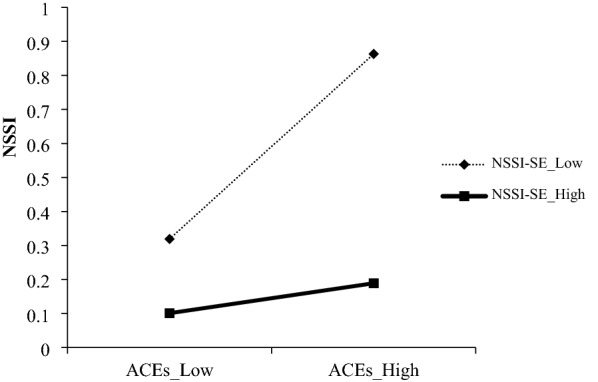
Self-efficacy moderated the association between ACEs and NSSI

**Fig. 3:**
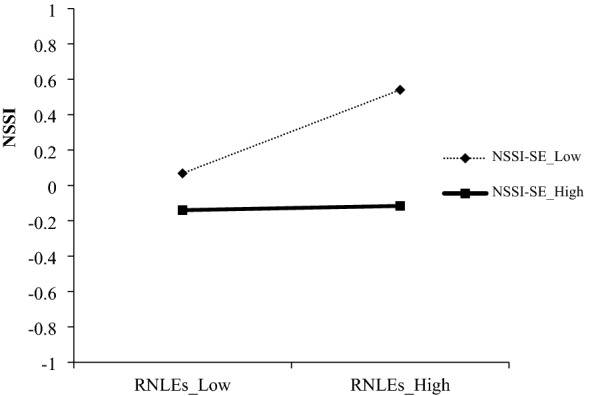
Self-efficacy moderated the association between RNLEs and NSSI

To study the interaction between ACEs and NSSI-SE, a slope test was carried out. Figure 2 shows a significant positive association between this group of ACEs and NSSI when in conjunction with low (− 1 standard deviation) levels of NSSI-SE (*B* = 0.27, *t* = 8.53, *p* < 0.001) but not high (+ 1 standard deviation) levels (*B* = 0.04, *t* = 1.38, *p* > 0.05). Then, a slope test was performed to explore the interaction between RNLEs and NSSI-SE. As shown in Fig. 3, a significant positive association was found between this group of RNLEs and NSSI when in conjunction with low (− 1 standard deviation) levels of NSSI-SE (*B* = 0.24, *t* = 7.23, *p* < 0.001) but not high (+ 1 standard deviation) levels (*B* = 0.01, *t* = 0.37, *p* > 0.05). Thus, the findings are in line with previous hypotheses about the protective effects of NSSI-SE.

## Discussion

Based on the high prevalence of NSSI among college students and theories related to this phenomenon, the present study adds to our knowledge by examining the relationship between adverse childhood experiences (ACEs), recent negative life events (RNLEs), and NSSI among college students and exploring the protective effect of self-efficacy (NSSI-SE) on these relations. Our results provide a new perspective for the further prevention of NSSI among college students.

First, our research shows that ACEs have been strongly associated with NSSI among college students, which indicates that adverse childhood experiences significantly increases the risks of engaging in NSSI [71]. As shown by previous studies, childhood trauma destroys an individual’s ability to appropriately regulate or manage negative emotional states, which may further create significant emotional problems and extreme distress for individuals [13, 21]. As a result, individuals may use self-injury as a regulatory means of dealing with distress disorders that disturb cognitive and affective processing. Second, higher rates of RNLEs were significantly positively associated with more NSSI. The finding is consistent with, and builds upon, previous multi-wave and longitudinal studies examining the relationship between these two variables [72, 73]. This implies that both childhood and recent stressors should be considered when making risk evaluations as well as developing prevention and intervention measures to prevent individuals exposed to adversity from engaging in NSSI. Third, our results provide support for the independent effect hypothesis of ACEs and RNLEs in affecting NSSI [37]. Given that adverse childhood experiences and recent stressors were not found to interact with NSSI, further research can investigate whether stressors from childhood and recent periods have a cumulative effect on NSSI [74].

Our study is the first to examine the protective role of NSSI-SE in the relationship between ACEs, RNLEs, and NSSI among the targeted demographic of Chinese college students. The results show that for individuals exhibiting high levels of NSSI-SE, ACEs and RNLEs cannot significantly predict NSSI, which means that ACEs and RNLEs are positively associated with NSSI only in college students with lower levels of NSSI-SE. This means that NSSI-SE reduces the risk of persons with a history of ACEs and RNLEs engaging in NSSI. From the perspective of the TTM, NSSI-SE could be recognized as the ability to resist NSSI [40, 41]. Individuals with a high level of NSSI-SE have the potential to engage in positive self-suggestion when faced with stressful events and to believe that they can engage in appropriate coping interventions to reduce stress, thus avoiding negative coping strategies such as NSSI. Therefore, high NSSI-SE constitutes a significant protective factor for undergraduates in resisting NSSI whether they have suffered stimuli from distal or proximal stressors. The protective effect of NSSI-SE for individuals with a history of ACEs or RNLEs is a significant finding of this research, as NSSI-SE may prove to be an important protective factor for the improvement of therapy to help patients with a history of high risk-seeking avoid engaging in NSSI.

The results of this study have important implications for intervention in NSSI among college students. The results suggest a need to pay attention to college students who have experienced a series of stressors and highlight the importance of improving levels of NSSI-SE as a means to mitigate the effects of ACEs and RNLEs on NSSI. Enhancing NSSI-SE may be an important step toward equipping individuals with a history of ACEs and RNLEs to handle high-risk emotional situations in the college environment. In response to this finding, we should pay attention to improving NSSI-SE in college mental health programs and adding evaluations of relevant indicators to annual student mental health surveys.

Although the results of this research work have important value for clinical intervention in NSSI among college students, several limitations should be considered. First, cross-sectional data were acquired at a single time point, limiting our ability to monitor changes in the relationships of variables across time. Therefore, a longitudinal design is needed to confirm the relationships between these variables over time. Second, we examined ACEs and RNLEs in general, while different types of stressful experiences associated with NSSI may represent different mechanisms; therefore, future research should consider the specific effects of different stressful events relating to NSSI. Third, based on the larger proportion of female participation in our survey, the design of our survey should be improved to increase male participation to ensure a more equal gender response rate and reduce the likelihood of self-selection to obtain more accurate data on gender differences in NSSI.

## Data Availability

The datasets analyzed in the current study are available from the first author and corresponding author on reasonable request.

## Abbreviations

ACEs: Adverse childhood experiences
RNLEs: Recent negative life events
NSSI-SE: Self-efficacy to avoid non-suicidal self-injury
NSSI: Non-suicidal self-injury
